# Let the Avatar Brighten Your Smile: Effects of Enhancing Facial Expressions in Virtual Environments

**DOI:** 10.1371/journal.pone.0161794

**Published:** 2016-09-07

**Authors:** Soo Youn Oh, Jeremy Bailenson, Nicole Krämer, Benjamin Li

**Affiliations:** 1 Department of Communication, Stanford University, Stanford, CA, United States of America; 2 Department of Computer Science and Applied Cognitive Science, Social Psychology: Media and Communication, University of Duisburg-Essen, Duisburg, Germany; University of Pécs Medical School, HUNGARY

## Abstract

Previous studies demonstrated the positive effects of smiling on interpersonal outcomes. The present research examined if enhancing one’s smile in a virtual environment could lead to a more positive communication experience. In the current study, participants’ facial expressions were tracked and mapped on a digital avatar during a real-time dyadic conversation. The avatar’s smile was rendered such that it was either a slightly enhanced version or a veridical version of the participant’s actual smile. Linguistic analyses using the Linguistic Inquiry Word Count (LIWC) revealed that participants who communicated with each other via avatars that exhibited enhanced smiles used more positive words to describe their interaction experience compared to those who communicated via avatars that displayed smiling behavior reflecting the participants’ actual smiles. In addition, self-report measures showed that participants in the ‘enhanced smile’ condition felt more positive affect after the conversation and experienced stronger social presence compared to the ‘normal smile’ condition. These results are particularly striking when considering the fact that most participants (>90%) were unable to detect the smiling manipulation. This is the first study to demonstrate the positive effects of transforming unacquainted individuals’ actual smiling behavior during a real-time avatar-networked conversation.

## Introduction

Cyberspace was envisioned to bring “an age of affordable beauty” [1]^p3^ where transformations to the self would be effortless. Within this space, people would be able to freely change their appearance and behavior in ways that best fit their goals. Researchers hypothesized that individuals would be able to construct and reconstruct their identities through computer-mediated communication [2]. Indeed, a brief look at the current online sociosphere appears to confirm these early prophesies. People strategically manage their posts on social media to promote desirable aspects of themselves [3]. Individuals even misrepresent certain physical aspects of their actual selves, such as height or weight, on online dating websites to present themselves in a more positive light [4]. According to Goffman, this desire to manage and control how other people perceive and subsequently treat the self is thought to be a part of everyday interactions [5]. In ‘performing the self’, individuals aim “to convey an impression to others which it is in his interests to convey” [5]^p4^.

Walther’s hyperpersonal model of communication suggests that people are often able to present themselves in a more positive light via computer-mediated communication (CMC) than during a face-to-face (FtF) conversation, because they have the time to strategically select the information that they wish to disclose about themselves [6]. The model further proposes that these effects are particularly stronger for asynchronous CMC, as this form of communication grants people more time to monitor and edit the messages that they wish to send to the other person. Underscoring this argument, Toma, Hancock, and Ellison posit that online daters are able to ‘reallocate’ cognitive resources into creating a desirable dating profile in lieu of trying to manage their verbal and nonverbal behavior in a real-time face-to-face conversation [7].

Indeed, one of the reasons CMC was thought to yield hyperpersonal relationships was due to the relative absence of nonverbal leakages that may lead to undesirable interpersonal consequences [7]. While nonverbal cues are not necessarily linked with the communicator’s actual feelings or truthfulness (see [8] for review), people tend to rely on these nonverbal behaviors to detect deception and/or form interpersonal judgments [9–11]. Despite the significance of nonverbal cues in interpersonal interactions, controlling nonverbal behavior during a real-time conversation has been difficult to do in both offline and online contexts.

Recent advancements in virtual reality technology, however, have led to the possibility of strategically controlling and altering one’s nonverbal behavior in computer-mediated contexts (i.e., transformed social interaction) [12]. Multiple studies have explored the interpersonal outcomes of transforming nonverbal cues of virtual humans [13–16]. For example, [14] found that people who interacted with an avatar that had been programmed to engage in a long mutual gaze (4 seconds) with them felt more positively toward their partner than when the mutual gaze was short (2 seconds). However, with a few notable exceptions [14], [17–18], most of these studies explored the influence of computer-controlled *agents*’ nonverbal behavior and not that of human-controlled *avatars*. Furthermore, even studies that did examine the implications of *avatar*’s nonverbal behavior often used computerized agents that were introduced to participants as avatars (e.g., [19]). While this measure was necessary in order to maximize experimental control, it also imposed restrictions in terms of ecological validity, as the conversational content between the ‘avatar’ and participant was fairly limited.

Avatars are visual representations of their users in virtual environments and can be separated from agents by the source of control; avatars are controlled by actual people, while agents are controlled by computerized algorithms [20]. Due to this difference in agency, (perceived) avatars and agents often yield different levels of social influence. For example, Hoyt and colleagues [21] conducted a study wherein participants were asked to perform a learned or novel task in front of virtual humans. Despite the fact that these virtual humans were not representations of actual people, they found that participants’ performance of the novel task was impaired when they were led to believe that the virtual audience consisted of avatars, but not when they were framed as agents. In their recent meta-analysis, [22] also concluded that avatars exert stronger levels of social influence compared to agents. As such, the current literature on virtual humans’ nonverbal cues would greatly benefit from studies that incorporate avatars that are controlled by actual people.

Among other nonverbal signals, a “substantial body of evidence…indicates that facial expressions serve as rich nonverbal cues that can powerfully communicate important interpersonal intentions and motives” [23]^p730^. Out of these facial expressions, smiling commonly indicates high levels of social interest and acceptance [23] and plays a special role in interpersonal communication [24]. Studies show that smiling is used as a way to form bonds with others [25] and that smiling behavior is more pronounced when people are engaged in social contact [26].

Perhaps because smiling signals positive interpersonal intensions, research consistently shows positive effects of smiling on communication outcomes; people feel happier and smile more in the presence of people whose facial expressions indicate happiness (i.e., smiling); in addition, smiling people are often given more positive interpersonal ratings than people with neutral expressions [27–28]. Krumhuber and colleagues [29] found that even people who exhibit a smile that is not perceived as genuine are rated more likeable, attractive, trustworthy, and cooperative compared to people who assume a neutral expression. However, targets with genuine smiles still received the most positive ratings.

Despite the significant interpersonal function of smiling, most of the research on avatar nonverbal behavior has been limited to the movement of the neck (and resulting head position and rotation) and eyes. Although some studies explored the influence of a smiling avatar or agent on interpersonal perceptions (e.g., [24], [30–32]), results were mixed; technological limitations may have hampered the virtual human’s smiling behavior, leading to low behavioral realism (e.g., static smile, intermittent transitions from neutral to smiling expression). In one study, Cafaro and colleague [30] had participants walk up to a smiling or non-smiling virtual agent in an immersive virtual environment, and found that participants judged the agent more positively when it was smiling. In contrast, Krämer et al. [24] found that while interacting with a virtual agent that smiled frequently led to an increase in smiling behavior, it did *not* improve ratings of the virtual agent. Verhagen and colleagues [31] used smiling and non-smiling static images to represent a virtual customer agent, and did not find any differences in evaluations based on smiling behavior. In addition to the inconclusive results of the aforementioned studies, it is also important to note that all of these studies were conducted using computerized agents, and not avatars controlled by actual people.

One study that did compare the effects of smiling behavior between agents and avatars found that while smiling agents received more positive evaluations than non-smiling agents, smiling behavior ironically led to more negative evaluations when participants were told that they were communicating with a real person [32]. These results are surprising considering the wealth of literature that point to the positive outcomes of smiles. While it is likely that there is more than one explanation for this unexpected finding, the authors suggest that one reason for these results was the limited technology at the time [32], wherein the experimenter activated a pre-programmed smile by pressing a button. The level of realism provided by such a smile may not have been sufficient for the relatively higher thresholds of behavioral realism people have for actual humans compared to computerized agents.

With the rapid advancement of facial tracking technology in the past few years, it is now less challenging—both computationally and financially—to render realistic facial expressions on an avatar. A number of new technologies allow virtual environments to use facial tracking data from webcams or specialized depth cameras to render the user’s actual expressions on a virtual character in real-time (e.g., *Face Analysis*, *FaceRig*, *Faceshift*, *Faceware*, *Nito*, and *Pocket Avatars*). More importantly, the interactants in virtual environments can now subtly alter and enhance these expressions. For instance, considering the positive social implications of smiling, the user can choose to enhance his or her avatar’s smile. This approach differs from that of previous smiling research in that it uses the interactant’s actual behavior as a baseline, instead of using a pre-programmed smile that is activated on a button press. If the user doesn’t smile at all, the avatar would not smile either; it is only when the user actually smiles that the avatar exhibits (enhanced) smiling behavior. Such an approach is more likely to allow for a more realistic transition between facial expressions as well as a higher consistency between verbal and nonverbal behavior. As such, the current advancement in technology offers researchers the possibility to study the implications of nonverbal cues in both a more controlled *and* practically relevant manner.

## The Present Study

The primary aim of the study is to determine if ‘enhanced’ (more intense) smiles would yield more positive communication outcomes compared to ‘normal’ (consistent with participants’ actual) smiles. More specifically, we were interested in if enhancing the smiles of the interactants’ avatars would lead participants to feel greater positive affect and like their partner more. In addition, we were curious how enhancing the avatar’s smile would influence participants’ perception of their communication experience, namely social presence. Social presence refers to “the sense of being with another” [33]^p456^ and is used to assess the extent to which communicators are aware of the presence of another person and have access to the other person’s affective and cognitive states [34]. Higher social presence is generally associated with higher satisfaction with the communication medium. Finally, we explored if participants in the enhanced smile condition would choose to spend more time on the avatar-networking platform (vs. FtF communication) when given the option to divide their time between different modalities. We hypothesized that communicating via avatars that ‘enhanced’ their actual smiles would yield more positive interpersonal outcomes than avatars that conveyed participants’ actual smiles on all three interpersonal measures (i.e., participants’ positive affect, attraction toward their partner, social presence of their partner). We also hypothesized that participants in the ‘enhanced smile’ condition would want to spend more time on the avatar-networking platform (vs. FtF communication) than those in the ‘normal smile’ condition.

Second, we wanted to see if there were benefits of adding realistic facial expressions to an avatar compared to providing an avatar with a narrow range of facial movements (i.e., mouth that only opens and closes). The extant literature suggests that increasing behavioral realism yields a more positive communication outcome [35–37]. For example, von der Pütten and colleagues [37] found that participants felt higher levels of mutual awareness when they spoke with a virtual human that blinked, shifted posture, breathed, and nodded in response to the participant (back-channeling) compared to a virtual human that did not exhibit back-channeling behavior. However, the implications of adding realistic facial expressions to an avatar are yet to be explored. As such, we included an avatar that did not exhibit any facial expressions except for the opening and closing of the mouth (i.e., ‘mouth open-close’ condition) along with avatars that offered either a veridical or enhanced version of the participant’s smile in our study design (i.e., ‘normal smile’ and ‘enhanced smile’ conditions).

In summary, the following hypotheses and research question was proposed:

*Hypothesis 1a-d*: Participants in the enhanced smile condition will feel greater (a) positive affect, (b) attraction toward their partner, (c) social presence of their partner and (d) desire to spend more time communicating via the avatar-networking platform compared to those in the normal smile condition.
*Research Question1a-d*: Does the addition of realistic facial expressions influence participants’ (a) positive affect, (b) attraction toward their partner, (c) social presence, and (d) desire to spend time communicating via the avatar-networking program?

## Method

### Participants

A total of 158 participants (54 men, 104 women) were recruited from a medium-sized western university. Of these, 81 were White, 29 were Asian, 17 were Black, 12 were Latino, and 19 reported another ethnicity. The mean age of the participants was 20.96 (SD = 2.81). All participants were assigned to same-gender dyads to account for potential gender effects.

### Ethics Statement

All procedures and materials were approved by the Ethical Committee of the Institutional Review Board at Stanford University. Written consent was obtained from all participants.

### Apparatus

We used a markerless motion capture software with a frame rate of 30 frames per second. The software used was *Faceshift*, which uses a Facial Action Coding System solver to categorize facial motions into 51 unique expressions (e.g., open mouth, smile, etc.) and tracks the extent to which each expression is activated [38]. A depth camera that uses infrared sensors and adaptive depth detection (*Asus* Xtion PRO Live) was used to track facial expressions in addition to the translation (x, y, z) and orientation (pitch, yaw, roll) of the participant’s physical head and eyes. This tracking data was then used to update the avatar’s head, eyes, and facial expressions. The *Unity* game engine was used to generate and program the avatar interaction platform.

### Study Design

The present study adopted a dyadic design with three conditions: Normal Smile, Enhanced Smile, and Mouth Open-Close only. Both participants in the dyad were assigned an avatar with identical smiling conditions (e.g., participants in the enhanced smile condition both had avatars that enhanced their actual smiles), although they were only able to see their partners’ avatars.

In order to determine the extent to which the smile should be enhanced in the Enhanced Smile condition, we ran a pilot study with 23 participants who were not a part of this study. The coefficient values of the smile tracking data were initially multiplied by 1.2 when updating the avatar’s smile in the enhanced smile condition. However, participants provided feedback that the smiling behaviors of the enhanced and normal smile conditions were similar. Thus, we increased the enhancement of the smiling coefficients to 1.5, which did lead to perceptions that the avatars in the enhanced smile condition smiled more than the normal smile condition. More importantly, post-pilot interviews with participants showed that they had not been aware of the smiling manipulation and thought that the conversational flow was natural. Based on these results, we chose to multiply the smiling data by 1.5; manipulation checks that were conducted after the actual study also confirmed the validity of this choice.

**Fig 1:**
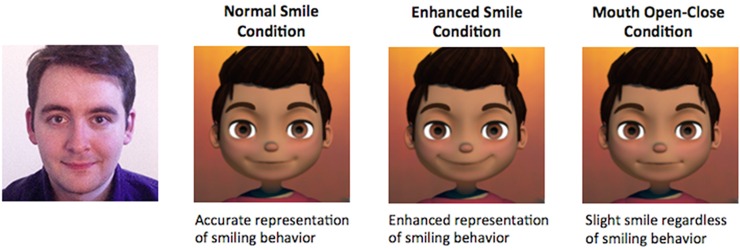
Avatar representation of ‘normal smile’, ‘enhanced smile’, and ‘mouth open-close only’ conditions.

In contrast, only the coefficient value that represented the extent to which a mouth was open was used to update the avatar in the mouth open-close condition. To allow for a fair comparison across the three avatars, the avatar in the mouth open-close only condition was designed to exhibit a slight smile. The different avatar representations of these conditions is available in Fig 1.

### Materials

**Fig 2:**
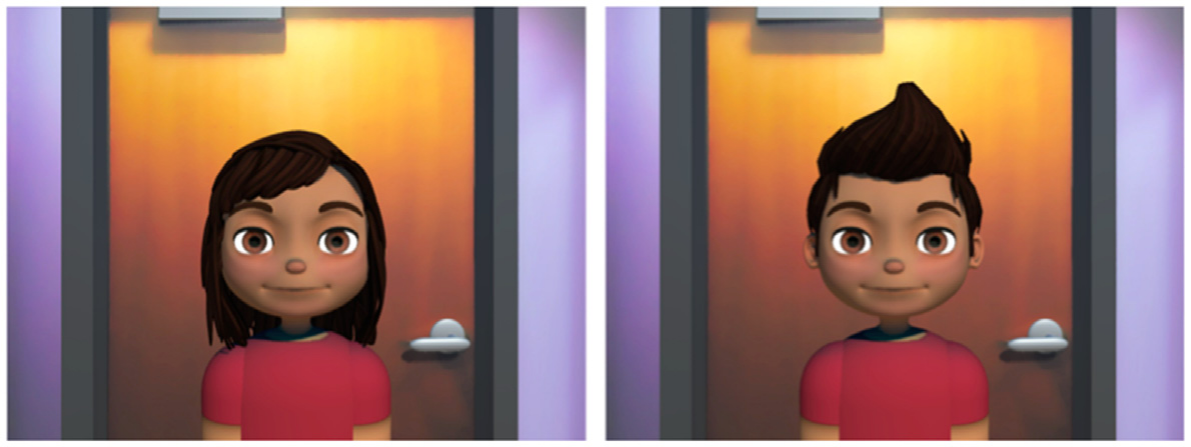
Neutral state female (left) and male (right) avatars used in study.

All participants were represented by avatars that matched their (biological) genders. The background of the virtual interaction platform was modeled after the room in which the participants were seated. The features of both the female and male avatars were similar to each other with the exception of the hairstyle (Fig 2). Participants only saw their partners’ avatars, and not their own during the interaction. All female participants were given the same female avatar, and all male participants were assigned the same male avatar.

### Procedure

Dyads were randomly assigned to one of the three conditions (Normal Smile, Enhanced Smile, Mouth Open-Close only). Participants were asked to come to a different location prior to the study to prevent them from interacting with their partner prior to the virtual interaction. Participants who reported that they knew each other (n = 6 individuals) after the interaction were excluded from analysis.

Upon given consent to participate in the experiment, participants were told that they would communicate with a partner via an avatar-networking program. The experimenter then explained that a facial tracking program (*Faceshift*) would be used to scan five expressions (i.e., neutral, open mouth, smile, pressing lips together, puffing out cheeks) that would be used to create a personalized tracking profile. The tracking profile allows the facial tracking program to understand how each participant’s exhibits a specific facial expression, and to detect the position of his or her facial features. After scanning these expressions and confirming that the program was tracking the expressions accurately, the experimenter told the participants that they would be playing a 20 Questions Game with their partner. The objective of the 20 Questions Game is to guess the partner’s word by asking 20 or fewer questions. Participants were each assigned one of the following words: “rabbit” or “ocean”.

After receiving these instructions, participants were able to see and hear each other via the avatar interaction platform. Participants were *only* able to see their partner’s avatar, and were *not* able to see their own avatars. Participants spoke into microphones during their conversations, and were able to hear each other through headphones provided. Participants’ facial movements were tracked and mapped in real-time on their avatar on their partner’s screens, allowing for their mouths to move when they were speaking. As noted before, depending on the condition, the smile was either enhanced (‘enhanced smile’) or the range of mouth movements was limited (‘mouth open-close only’). Once confirming that they could see and hear each other, participants followed the instructions on the screen, which gave them instructions regarding the 20 Questions Game. After the game, participants were asked to spend ten minutes to get to know their partner (‘Getting to Know You’ task). Participants were told that they could divide their time between the avatar-networking program and in-person (face-to-face) communication. They were asked to let their partner know if, at any point during the second task, they wished to speak in person. They were also told that both would have to come to a mutual agreement prior to changing the interaction modality. Participants were thus able to engage in a discussion regarding whether or not they wished to speak in person. At the point in time when participants either (1) agreed that they would like to speak face-to-face or (2) spent the entire 10 minutes interacting via the avatar-networking program, they answered a questionnaire on a computer via *Qualtrics* (after which they met their partners). All questions were presented in the same order (see *Measures* for information about questions). The amount of time participants chose to spend on the avatar-networking platform was recorded. Essentially, participants’ head and mouth movements were tracked, captured and rendered on the avatars either with or without digital manipulations, depending on the condition. The smile was manipulated to be enhanced in the ‘enhanced smile’ condition and the range of mouth movements was manipulated to be limited to open-close movements only in the ‘mouth open-close only’ condition. Facial tracking data were not manipulated in the ‘normal smile’ condition. Participants’ voices, the content of their conversations and the point that they decide to speak face-to-face were free from manipulation.

### Measures

#### Manipulation checks

In order to assess if the smiling manipulation had been successful, participants were asked how strongly they agreed with the following statement: “My partner's avatar smiled during our conversation” on a 7 point Likert-type scale (1 = *Strongly Disagree*, 7 = *Strongly Agree*). Greater values indicate stronger agreement (*M* = 5.54, *SD* = 1.32). The minimum was 2 and the maximum was 7.

In addition, to assess if participants perceived the avatars with realistic facial expressions to be more realistic than the avatars in the mouth open-close only condition, participants were asked to indicate their level of agreement with the following: “My partner's avatar's mouth movements were realistic during our conversation”. Greater values indicate stronger agreement (*M* = 4.06, *SD* = 1.61). The minimum was 1 and the maximum was 7.

#### Positive and negative affect

Affect was measured using the Positive and Negative Affect Schedule (PANAS) [39]. Participants were given 20 different feelings and emotions (e.g., interested, distressed, excited) and were asked the extent to which they felt each one at the present moment on a 5-point Likert-type scale (1 = *Not at all*, 5 = *Extremely*). The order in which the positive and negative emotions words were presented was randomized. The scale was good for positive affect terms, Cronbach’s α = .89 (*M* = 2.72, *SD* = .74) and acceptable for negative affect terms, Cronbach’s α = .72 (*M* = 1.26, *SD* = .28); the minimum was 1.2 and 1 for positive and negative affect respectively. The maximum were 4.7 and 2.4 for positive and negative affect respectively.

#### Interpersonal attraction

Interpersonal attraction was measured with six items used in [40]. Sample items include “I would enjoy a casual conversation with my partner” and “I would get along well with my partner”. Participants were asked to rate how strongly they agreed with each statement on a 7 point Likert-type scale (1 = *Strongly Disagree*, 7 = *Strongly Agree*). The reliability of the scale was good, Cronbach’s α = .89. Greater values indicate stronger social presence (*M* = 5.65, *SD* = .76); the minimum was 3.5 and the maximum was 7. The wording for all of the above measures can be found in S1 Appendix.

#### Social presence

Social presence was measured by adapting items from the Networked Minds Social Presence Inventory [41] and Nowak and Biocca’s social presence measure [42] that were relevant to the current context. Items were slightly modified to fit the current context. Sample items include “I felt like I was in the same room as my partner” and “My partner’s emotions were clear to me”. Participants were asked to rate how strongly they agreed with each statement on a 7 point Likert-type scale (1 = *Strongly Disagree*, 7 = *Strongly Agree*). The reliability of the scale was good, Cronbach’s α = .80. Greater values indicate stronger social presence (*M* = 4.72, *SD* = .83); the minimum was 2.63 and the maximum was 6.38.

#### Linguistic cues of affect

To include a more behavioral measure of affect, participants were asked to describe their experience using the avatar-networking program in an open-ended format. These responses were analyzed using the Linguistic Inquiry and Word Count dictionary (LIWC) [43]. More specifically, they were asked, “Describe your experience talking with your partner using the avatar-networking program. Focus on how you felt during the interaction. Please be as detailed as possible.” Participants were asked to write at least five sentences. LIWC has been used as a reliable measure in various contexts, including the prediction of deception [44], personality [45], and emotions [46]. For our purposes, we only used the ‘affect’ category (positive and negative emotions) to analyze participant responses. Theoretically, each participant’s response could comprise of 100% emotion words, although such sentences are highly improbable. On average, 3.66% of a participant’s response was composed of positive emotion words (*SD* = 2.39; minimum = 0, maximum = 11.76). In addition, 1.39% of a participant’s response was composed of negative emotion words (*SD* = 1.35, minimum = 0, maximum = 6.82).

#### Smile manipulation detection

To check if participants detected the facial expression manipulation, we asked participants what they thought the purpose of the study was. Two independent coders examined these open-ended responses to determine if participants detected the manipulation (Cohen’s κ = .66). Participants who were judged by one or both of the raters to have expressed suspicion that the facial expressions had been altered in some way or indicated that the avatar looked happy were considered to have detected the smile manipulation. A total of 4 participants (all in the enhanced smile condition) were thus removed.

#### Preference for avatar-mediated platform (time)

To measure participants’ preference for the avatar-mediated platform compared to face-to-face interactions, we gave participants a ‘Getting to Know You’ task, and asked them to press a keyboard button when they wished to stop interacting via the avatar-mediated platform and talk face-to-face instead. Participants were asked to reach a mutual agreement regarding when they wished to meet each other during their conversation. Once one participant suggested talking face-to-face, the other participant could either agree or express his or her desire to continue the conversation on the avatar-mediated platform. Once both participants agreed that they wished to talk face-to-face, they each pressed a keyboard button at the same time, which ended their avatar-mediated communication. As mentioned previously, we measured the time it took participants to decide that they would rather talk face-to-face than use the avatar-mediated platform. Time was measured in milliseconds and later converted to minutes. If participants did not wish to switch platforms (i.e., spent the entire 10 minutes on the avatar-mediated platform), the interaction ended automatically when the 10 minutes had passed (*M* = 4.78 *SD* = 3.42); the minimum was 0.19 and the maximum was 10.

## Results

As mentioned earlier, six of the participants were excluded from analysis as they reported that they already knew each other (n = 6). In addition, due to a technological failure, participants in one dyad were assigned to different conditions (n = 2) and were thus excluded from analysis. Finally, the four participants that were thought to have guessed the purpose of the study and one of the participants who later reported not being able to see her partner were excluded from analysis (n = 5), leaving a total of 145 participants (n = 50, 46, and 49 for the normal smile condition, enhanced smile condition, and mouth open-close condition, respectively). Chi-squared tests showed equal distribution of gender (*χ*² = 3.905, *df* = 2, *p* = .14) and ethnicity (*χ*² = 10.99, *df* = 10, *p* = .36) across conditions. Age was also well distributed across the ‘normal smile’ (*M* = 20.86, *SD* = 2.17), ‘enhanced smile’ (*M* = 21.57, *SD* = 2.08) and ‘mouth open-close only’ (*M* = 20.35, *SD* = 3.87) conditions, with no significant differences between conditions (*F*(2, 142) = 2.26, *p* = .11). The means and standard deviations for all of the dependent variables by condition can be seen in Table 1.

**Table 1:** Means and standard deviations of dependent variables by condition.

	Normal Smile	Enhanced Smile	Mouth Open-Close Only
	*M (SD)*	*M (SD)*	*M (SD)*
Positive Affect	2.57 (.81)	2.95 (.63)	2.62 (.66)
Negative Affect	1.34 (.32)	1.22 (.23)	1.24 (.30)
Attraction	5.48 (.81)	5.80 (.69)	5.60 (.73)
Social Presence	4.50 (.79)	4.95 (.82)	4.66 (.77)
LIWC (Positive)	2.99 (1.96)	4.31 (2.57)	3.59 (2.39)
LIWC (Negative)	1.49 (1.47)	1.47 (1.37)	1.24 (1.23)
Time Spent	4.47 (3.75)	4.97 (3.04)	5.17 (3.39)

Data from participant who interact with each other are likely to violate the assumption of independence and is thus inappropriate for ANOVA and regression approaches [47]. Multilevel analysis (also known as mixed-effects model, hierarchical linear model, etc.) “combines the effects of variables at different levels into a single model, while accounting for the interdependence among observations within higher-level units” [48]^p3665^. As neglecting intragroup dependence can bias statistical estimates, we used a multilevel model to address this issue (for an example of a study that uses this model, see [49]). We used the ‘nlme’ package in R to conduct a linear mixed-model analysis. We accounted for the random effects that arise from the individual subjects who are nested within dyads and used compound symmetry structure for the within-group correlation structure. The different conditions were dummy-coded and treated as a fixed factor (avatar facial expression) with three levels (i.e., normal smile, enhanced smile, mouth open-close only). Gender was included as a control variable to account for the possible gender differences in smiling behavior and perception [50]. Other studies that study the influence of nonverbal cues have included gender as control variables when they were considered relevant [17], [51]). All analyses below are results from this linear mixed-effects model, with the exception of the impact of avatar condition on the time spent in the avatar-networking program (in this case, we used a multiple regression model that used the dyad as the unit of analysis; this was because partners mutually reached the time decision during their conversation and, as such, there was a single data point per dyad) and the correlational analyses. Results from the linear mixed-effects analyses are reported with an estimate value (*b*) and significance level (*p)*, which is in line with studies that used multilevel models [52–53].

### Manipulation Checks

A manipulation check controlling for gender confirmed that participants in the enhanced smile condition agreed more strongly with the statement that their partner’s avatar smiled (*M* = 5.24, *SD* = 1.35 vs. *M* = 6.43, *SD* = .83; *b* = 1.20, *p*< .001). In contrast, there was no difference between the normal smile condition and mouth open-close only condition (*M* = 5.24, *SD* = 1.35 vs. *M* = 4.94, *SD* = 1.28; *b* = -.30, *p* = .25). This is not surprising, considering that the mouth open-close only avatar was designed to have a slight smile. In addition, participants in the mouth open-close only condition perceived the movements of their partner’s avatar to be less realistic than the normal smile conditions (*M* = 4.17, *SD* = 1.51 vs. *M* = 3.47, *SD* = 1.47; *b* = -.74, *p*< .05). In contrast, there was no difference in perceived realism between the normal smile and enhanced smile conditions (*M* = 4.17, *SD* = 1.51 vs. *M* = 4.52, *SD* = 1.77; *b* = .33, *p* = .38). Due to a technical error, the manipulation check data for *perceived realism* is missing for six dyads (n = 12).

### Positive Affect

Participants in the enhanced smile condition reported feeling more positive affect compared to the normal smile condition (*M* = 2.95, *SD* = .63 vs. *M* = 2.57, *SD* = .81, *β* = .22, *p*< .05), confirming *Hypothesis 1a*. In contrast, there were no statistically significant differences in positive affect between the normal smile condition and mouth open-close condition (*M* = 2.57, *SD* = .81 vs. *M* = 2.62, *SD* = .66, *β* = .05, *p* = .57), *Research Question 1a*. In terms of our control variable, males reported higher levels of positive affect compared to females (*M* = 3.00, *SD* = .72 vs. *M* = 2.58, *SD* = .69, *β* = -.23, *p*< .01), which is consistent with some previous findings that males tend to report more positive feelings than females (e.g., [54]).

### Negative Affect

There was no significant difference in negative affect between the enhanced smile and normal smile conditions (*M* = 1.22, *SD* = .2 vs. *M =* 1.34, *SD* = .32, *β = -*.*20*, *p* = .06). Similarly, significant differences were not found between the normal smile and mouth open-close only conditions (*M* = 1.34, *SD* = .32 vs. *M* = 1.24, *SD* = .30, *β = -*.*16*, *p* = .14).

### Interpersonal Attraction

There was no significant difference in attraction between the enhanced smile and normal smile condition (*M* = 5.80, *SD* = .69 vs. *M* = 5.48, *SD* = .81, *β* = .21, *p* = .06). Similarly, interpersonal attraction ratings in the open-close only condition did not differ significantly from those in the normal smile condition (*M* = 5.48, *SD* = .81 vs. *M* = 5.60, *SD* = .73, *β* = .07, *p* = .54), *Research Question 1b*.

### Social Presence

Participants in the enhanced smile condition experienced higher levels of social presence compared to the normal smile condition (*M* = 4.95, *SD* = .82 vs. *M* = 4.50, *SD* = .79, *β* = .24, *p*< .05), supporting *Hypothesis 1c*. In contrast, participants in the mouth open-close only condition did not report different levels of social presence from those in the normal smile condition (*M* = 4.66, *SD* = .77 vs. *M* = 4.50, *SD* = .79, *β* = .06, *p* = .56), *Research Question 1c*. These results suggest that the addition of realistic facial expressions to an avatar do not automatically yield higher perceptions of social presence.

### Linguistic Inquiry & Word Count (LIWC)

Consistent with the PANAS data, we found that participants in the enhanced smile condition used a higher percentage of positive emotion words compared to the normal smile condition (*M* = 4.31, *SD* = 2.57 vs. *M* = 2.99, *SD* = 1.96, *β* = .27, *p*< .01), offering further support for *Hypothesis 1a*. In contrast, there were no statistically significant differences in the proportion of positive emotion words between the normal smile condition and mouth open-close condition (*M* = 2.99, *SD* = 1.96 vs. *M* = 3.59, *SD* = 2.39, *β* = .12, *p* = .24). *Research Question 1a*. No differences were found between conditions in terms of the proportion of negative words that were used (both *p*s> .30). Participants’ open-ended responses had an average word count of 95.7 (*SD* = 43.84). While this satisfies [43]’s suggestion of a minimum of 50 words per response, 14 responses comprised of less than 50 words each. However, even after excluding these 14 entries, the significance of the results and effect sizes were comparable (Proportion of positive emotion words: Normal smile vs. Enhanced smile: *β* = .26, *p* = .01 & Normal smile vs. Open-Close: *β* = .13, *p* = .88; Proportion of negative emotion words: Normal smile vs. Enhanced smile: *β* = .11, *p* = .32 & Normal smile vs. Open-Close: *β* = -.02, *p* = .83). These entries were therefore included in the final analysis.

### Time Spent on Avatar-Mediated Platform

Because dyads decided when they wanted to talk face-to-face together, there was only one data point available per dyad. As such, regression analyses were conducted with the avatar condition and gender as predictors to examine the effects of the avatar condition on the time dyads chose to spend on the avatar-networking platform. Contrary to the questionnaire data, enhancing the avatar’s smile did not increase the time participants decided to spend on the avatar-mediated platform over face-to-face interaction compared to the normal smile condition (*β* = .09, *p* = .52), *Hypothesis 1d*. Differences were also not found between the normal smile condition and mouth open-close only condition (*β* = .08, *p* = .55), *Research Question 1d*. The effect of gender was not significant (*β* = .16, *p* = .19).

To account for the possibility that the varying length of times spent on the avatar-networking platform during the ‘Getting to Know You’ task could influence the outcome variables, we also conducted separate analyses for all outcome variables controlling for this time. Controlling for time did not substantively influence the main effect of the avatar conditions for any of the dependent variables and resulted in a worse model fit when comparing AIC levels; thus our final analyses did not include this variable.

### Correlations Among Dependent Variables

All simple correlations among dependent variables are included in Table 2. Correlation analyses showed that the time spent on the avatar-mediated platform was positively correlated with partner attraction (*r* = .31, *p*< .001) and social presence (*r* = .26, *p*< .01) and negatively correlated with negative affect (*r* = -1.78, *p*< .05). While it is difficult to definitively determine causality from this data alone, these results suggest that participants may have preferred to spend more time on the avatar-mediated platform when they were having a positive experience. Correlation analyses also revealed a positive correlation between PANAS and the results of the LIWC analysis (positive affect: *r* = .27, *p*< .001; negative affect: *r* = .16, *p*< .05). Furthermore, the percentage of positive words used by participants was significantly correlated with positive perceptions of the communication experience, namely partner attraction (*r* = .29, *p*< .001) and social presence (*r* = .32, *p*< .001).

**Table 2:** Simple correlations among dependent variables.

	Positive Affect	Negative Affect	Attraction	Social Presence	LIWC (Positive)	LIWC (Negative)	Time Spent
Positive Affect		.12	**.48**^a^	**.41**^a^	**.27**^b^	**-.22**^b^	.13
Negative Affect			-.07	**-.17**^c^	**-.14**^d^	**.16**^c^	**-.18**^c^
Attraction				**.45**^a^	**.30**^a^	-.10	**.30**^a^
Social Presence					**.34**^a^	-.10	**.25**^b^
LIWC (Positive)						-.12	.08
LIWC (Negative)							-.07

^a^ positive at *p*< .001;

^b^ positive at *p*< .01;

^c^ positive at *p*< .05;

^d^ positive at *p*< .10

## Discussion

The present study found that participants who interacted with each other using avatars that enhanced their actual smiles felt more positive affect and a greater sense of being present with their partner (i.e., social presence). Furthermore, when asked to describe their experience using the avatar-networking platform, participants were more likely to use a greater proportion of positive words when they had been assigned to the enhanced smile condition, compared to the other conditions. Another important aspect to note is that the overwhelming majority of the participants were not aware of the smile manipulation.

To the best of our knowledge, this is the first study that examines if altering interactants’ facial expressions via real-time rendering on an avatar can influence one’s emotions, evaluations of their partner, and social presence. While there was a single study that did examine if altering interactants’ facial expressions during a video conference would impact creativity [55], the study’s participants were already acquainted with each other, making it difficult to tease apart the impact of the manipulation. Our study controlled for the familiarity factor by having participants interact with strangers. The results of our study offer strong evidence that enhancing certain facial expressions on avatar representation can influence interactants’ communication experience in virtual environments, at least among zero-acquaintance individuals. These findings support the rich literature on nonverbal cues in that increased smiling led to more positive outcomes [27–28], [56–57]. Our findings have practical implications for designers of virtual environments; introducing subtle changes in facial expressions may lead to a more positive experience between interactants. These findings are consistent with the literature on transformed social interaction [12], which show that virtual environments have the potential to allow people to strategically decouple their actual behavior and performance from their virtual representations to produce hyperpersonal communication outcomes that can be more positive than ‘authentic’ representations.

Contrary to our expectations, enhancing the smiling behavior of avatars did not influence the time participants preferred to spend on the avatar-networking platform over face-to-face communication. Considering the fact that participants evaluated their communication experience more positively for the increased smile condition, this is somewhat surprising. One possible explanation is that participants varied in their motives in selecting a communication modality. For instance, participants’ positive experience with the avatar-networking platform may have led to the desire to continue to communicate with their partner using the virtual platform, but this positive interaction experience could also have led to the eagerness to discover what their partners were like in ‘real life’. In addition, the content of the conversation itself may have also influenced participants’ willingness to switch interaction platforms. For instance, some of the participants may have encouraged their partners to meet in person more strongly than others.

Surprisingly, the normal smile condition did not yield additional benefits over the mouth open-close only condition. While we entertained the possibility that the increased realism of avatars with facial expressions that reflected facial motion tracking data would lead to positive communication outcomes, this was not the case. There are two possible explanations for this finding: first, because the avatar was not very high in terms of photographic realism (see Fig 2), participants may not have needed realistic facial expressions for a better communication experience. Studies suggest that avatars yield the most positive effects when the level of visual realism is congruent with the level of behavioral realism [58]. Second, it is possible that the nonverbal cues provided by the voice and up-and-down mouth movements may have been sufficient for simple interpersonal tasks (e.g., 20 Questions Game) between partners who were not required to see each other again. Previous research that compares audio and video conferencing also shows that adding video does not necessarily improve the communication experience [59].

## Limitations & Future Directions

Some limitations of the study need to be addressed. First we only used one type of avatar—one that had moderate levels of visual realism. While we chose this avatar to avoid confounds from ‘the uncanny valley effect,’ [60] wherein people feel negative emotions toward virtual representations that looks near-humanlike, it is possible that the appearance of the avatar will moderate the effects of enhanced smiles. Some studies on the perception of animated characters suggest that people perceived the same facial expressions to be less emotionally intense on more schematic faces (e.g., outline sketch) compared to real human faces [61]. Similarly, people considered artificially enhanced facial expressions to be less odd when the visual representation was a schematic sketch of the human face compared to when it was an actual human face [62]. In our study, the avatars were not very high in photographic realism, as they tended to look closer to a childish cartoon character rather than a ‘real’ human face. This may have added to the pleasantness of the interaction and influenced the potential effect of smiles on participants. Future studies should explore if and, if so, how the avatar’s appearance interacts with enhanced facial expressions.

In addition, our pilot study did not examine the quantitative relationship between the degree of smiling enhancement and perceptions when selecting the enhancement coefficient for the main study. As such, while we were able to select an enhancement coefficient that increased perceptions of smiling and still allowed the facial movements to appear natural (i.e., not manipulated), we cannot rule out the possibility that selecting another smiling coefficient through more quantitative testing may have yielded stronger effects. This is beyond the scope of the present study, but future studies should elucidate the relationship between the extent of smiling enhancement and perceptions.

Second, we focused specifically on zero-acquaintance dyads. While we did this to focus specifically on the effects of enhancing the avatar’s smile, it is possible that transforming nonverbal behaviors may have different implications depending on the relational dynamics between communicators. While they did not study this research question, [63] found that people smiled and laughed more and rated an audiotaped stand-up comedy routine more positively when they listened to canned laughter that they thought came from members of their in-group rather than their out-group. Studies have also shown that perceived group membership of the source influences people’s mimicry of nonverbal cues and empathy [64–65]. Future research could thus explore the boundary effects of enhancing smiles in virtual reality depending on the characteristics of the interactants’ relationship.

In addition to partners’ relationships with each other, the context of the conversation may also influence the effect of smiles. In the present study, participants were asked to play a 20 questions game and perform ‘Getting to Know you’ task in order to facilitate communication flow and allow for some control over the communication content. However, the relaxed and positive nature of a game may have yielded more smiles or fostered higher levels of attraction than a more serious task may have. Future studies should consider how the communication context can limit or enhance the influence of an avatar’s facial expressions.

In addition, our study was conducted in a social context, wherein participants were generally friendly towards one another. However, the increased smiles may be interpreted differently if the context was more hostile or competitive. In this case, enhanced smiles on an avatar may be perceived to be inconsistent with the interaction partner’s other behaviors (e.g., verbal), leading to poor communication outcomes. Studies have shown that people respond negatively to nonverbal behavior that is incongruent with verbal behavior for both real people and digitized agents, lending some support to this conjecture [66–67]. As such, more research is needed to determine when augmenting certain facial expressions yield positive outcomes, and when this is not the case.

It is also worth noting that the concept of social presence has often been linked to feelings of empathy. For instance, [68] proposed that empathy is a factor worthy of consideration in the psychological involvement dimension of social presence. The perception of being in the same space as another person has the potential to allow us to feel what that individual feels when something happens to her or him [69]. Virtual reality has the potential to allow people to step in the shoes of those whom they have not met before, or experience the lives of members of marginalized social groups. Future research can explore the links between avatar facial expressions, social presence and empathy, and examine how various aspects of virtual reality can contribute to a greater level of empathy among individuals.

Finally, our study participants were university students, and are thus more likely to have had experience interacting within virtual environments. However, as some studies suggest that age is associated with the level of social presence people feel in a virtual environment [70], it may be necessary to consider how different age groups as well as individuals with less experience with virtual environments respond to the different avatars before we are able to generalize our findings.

## Conclusion

The current results offer cogent evidence that enhancing the smile on one’s avatar can lead to more positive outcomes compared to when the smile is accurately mapped on the avatar. This is the first study to explore the outcomes of altering participants’ actual facial expressions in real-time on a computer-generated avatar using both self-report and behavioral measures. While the facial expression manipulation did not influence the time participants chose to spend on the avatar-networking platform or their smiling intensity, both of these measures were correlated with participants’ perceptions of their partners and social presence. This suggests that behavioral measures in virtual environments can be used as an unobtrusive measure that can predict people’s attitudes.

## Supporting Information

S1 Appendix Measures for dependent variables. (DOCX)

S1 Dataset Complete dataset for the present study. (PDF)
