# Supplementary material for: Let the Avatar Brighten Your Smile: Effects of Enhancing Facial Expressions in Virtual Environments
Source: PLoS One. 2016 Sep 7;11(9):e0161794. doi: 10.1371/journal.pone.0161794 (PMC5014416; doi:10.1371/journal.pone.0161794)
Supplement: S1 Appendix — (DOCX) [file pone.0161794.s001.docx]

**Appendix. Measures for Dependent Variables**

**Positive & Negative Affect**

This scale consists of a number of words that describe different feelings and emotions. Read each item and then select a dot from the scale below each word. Indicate to what extent you feel this way RIGHT NOW, that is, at the present moment (1= *Not at all*, 5= *Extremely*).

| **Positive Emotion Items** | **Negative Emotion Items** |
| --- | --- |
| Interested, Excited, Strong, Enthusiastic, Alert, Active, Proud, Inspired, Determined, Attentive | Distressed, Upset, Guilty, Scared, Hostile, Irritable, Ashamed, Nervous, Jittery, Afraid |

**Interpersonal Attraction**

How strongly do you agree or disagree with the following statements about your partner?

1. I like my partner
2. I would get along well with my partner.
3. I would enjoy a casual conversation with my partner.
4. My partner is the type of person I could become close friends with.
5. My partner is a good listener.
6. My partner is friendly.

**Social Presence**

How strongly do you agree or disagree with the following statements about your partner?

1. I felt like I was face-to-face with my partner.
2. I felt like I was in the same room as my partner.
3. I remained focused on my partner throughout our interaction.
4. My partner remained focused on me throughout our interaction.
5. My partner’s emotions were clear to me.
6. My emotions were clear to my partner.
7. My mood was influenced by my partner’s mood.
8. My partner’s mood was influenced by my mood.
