# Supplementary material for: Let the Avatar Brighten Your Smile: Effects of Enhancing Facial Expressions in Virtual Environments
Source: PLoS One. 2016 Sep 7;11(9):e0161794. doi: 10.1371/journal.pone.0161794 (PMC5014416; doi:10.1371/journal.pone.0161794)
Supplement: S1 Dataset — (PDF) [file pone.0161794.s002.pdf]

Dyad	PartnerID_within_Dyad	Condition (1=Normal, 2=Open/Close, 3=Enhanced)	PANAS_POS	PANAS_NEG	Attraction	SocialPresence	LIWC_positive	LIWC_negative	LIWC_WordCount	Time_in_ Milliseconds	Manipulation_Check_Smile	Manipulation_Check_Realistic	Gender	Race (1=White/Caucasian,2= African American, 3=Hispanic, 4=Asian, 5=Native American/Pacific Islander, 6=Other)
1	2	1	2.9	1.8	4.8333333	3.375	1.06	1.06	94	42745	3	3	2	1
1	1	1	2.5	1.1	4.5	4.375	1.18	1.18	85	42745	6	3	2	3
2	2	3	2.6	1.4	4	3.25	0	3.7	54	572798	6	6	2	1
2	1	3	2.7	2.2	4.8333333	4.125	2.22	0	90	572798	6	4	2	3
3	2	1	2.9	1.2	6	4.125	0.82	0.82	122	298489	6	4	2	4
3	1	1	2.4	1.9	5.1666667	5.25	5	2.5	80	298489	6	6	2	3
4	1	2	2.2	1.2	5.5	4.75	2.13	2.13	47	367311	5	2	2	4
4	2	2	1.6	1.2	6	5	0	2.34	128	367311	2	1	2	1
5	1	3	2.8	1.2	6	5.75	5.5	0.92	109	410542	7	6	2	4
5	2	3	2.1	1.1	5.6666667	4.5	2.54	1.69	118	410542	7	6	2	4
6	2	2	2.7	1.8	4.5	3.75	0	0	24	11226	3	2	1	4
6	1	2	1.4	1.2	3.8333333	4	2.56	1.28	78	11226	3	3	1	4
7	2	1	1.8	1	6	4	0	1.72	58	58744	5	2	1	1
7	1	1	1.4	1	4.6666667	4.625	3.96	0.99	101	58744	5	5	1	4
8	2	3	3.4	1.1	7	5.125	8.18	2.73	110	87151	7	7	1	6
8	1	3	3.4	1.4	5.5	4.5	0	0	33	87151	3	3	1	1
9	1	2	3	1	4.8333333	5.125	0	2.67	75	600000	4	2	1	2
9	2	2	2.5	1.2	6.1666667	5.625	3.31	1.1	181	600000	6	5	1	4
10	2	2	2.8	1.2	6	2.625	1.35	0	74	219971	6	3	2	1
10	1	2	1.9	1.1	6	4.5	6	0	50	219971	6	4	2	1
11	2	3	2.9	1.2	5.3333333	4.125	1.8	0.9	111	271281	6	2	2	4
11	1	3	2	1.1	5.6666667	5	8.54	0	82	271281	6	5	2	1
12	2	3	3.7	1.1	7	6.25	3.7	0	27	582098	6	2	2	2
12	1	3	3.2	1.2	7	5.875	2.76	0.46	217	582098	7	2	2	2
13	1	1	1.7	1	4	3.5	3.45	0	58	600000	5	2	2	2
13	2	1	4.6	1.5	6.6666667	5.25	7.75	0.7	142	600000	7	5	2	1
14	2	1	1.2	1	5.3333333	3.375	0	4.62	65	59254	6	6	2	2
14	1	1	1.8	1	5.3333333	5	4.05	1.35	74	59254	6	6	2	2
15	2	2	2.8	1	6.3333333	6.125	8.47	1.69	59	600000	5	5	2	4
15	1	2	1.8	1	5.8333333	4	1.71	2.56	117	600000	5	3	2	2
16	1	3	2.1	1.2	4.1666667	3.625	1.83	2.75	109	35157	6	1	2	3
16	2	3	2.6	1.1	5.1666667	5	2.38	1.59	126	35157	6	3	2	2
17	2	2	3.3	1.3	5.6666667	4.75	2.22	1.11	90	600000	6	6	2	1
17	1	2	1.6	1	6	5.125	3.26	4.35	92	600000	5	5	2	6
18	2	2	3.1	1.6	6.6666667	4.5	2.88	0.96	104	15628	5	4	2	1
18	1	2	2.1	1.2	5.1666667	4.625	0.89	0.89	112	15628	5	2	2	4
19	2	1	3.2	1.2	6.3333333	4.375	4.76	3.17	63	600000	6	NA	1	3
19	1	1	2.3	1.3	5.8333333	5.25	1.18	1.78	169	600000	6	NA	1	1
20	1	2	2.8	1.5	6	3.25	4.84	1.61	62	522968	6	5	2	1
20	2	2	3.3	1	6	5.75	4.39	0	114	522968	5	3	2	6
21	1	1	2.5	1.3	5.3333333	3.375	2.38	1.79	168	27927	3	3	1	1
21	2	1	1.8	1.5	5.5	3.25	3.18	1.86	377	27927	3	3	1	1
22	1	2	1.9	1	4.3333333	4.375	4.4	1.1	91	348977	3	5	2	1
22	2	2	3.2	1.2	5.8333333	5.625	4.88	1.22	82	348977	6	7	2	1
23	1	1	2.7	1.3	5.3333333	5.25	1.9	1.9	105	42234	2	6	2	1
23	2	1	1.5	1	5	3.25	2.83	0	106	42234	5	4	2	4
24	2	1	2.9	1.1	5.8333333	5.625	0	1.39	72	219974	6	5	2	1
24	1	1	2.5	1.5	6.1666667	4.375	1.65	1.65	182	219974	7	5	2	6
25	2	3	4	1.2	6.5	5.375	2.73	0.91	110	600000	7	6	2	1
25	1	3	1.9	1.1	5.3333333	4.875	2.27	0	44	600000	7	4	2	1
26	1	2	2.7	1	6.8333333	4.25	3.8	0	79	600000	6	3	2	2
26	2	2	3.1	1.4	7	5.75	4.69	2.34	128	600000	6	6	2	1
27	2	1	2.9	1.2	5.5	5.125	4.92	2.46	122	600000	7	5	2	3
27	1	1	2.1	1.7	4.8333333	4.375	0	4.69	64	600000	5	5	2	1
28	2	3	2.7	1.2	6.5	4.75	6.25	2.08	48	218685	7	2	1	1
29	1	1	3.8	2.1	6.5	5.25	3.67	1.83	109	82180	6	3	1	1
29	2	1	3.1	2.3	6	4.5	2.27	2.84	176	82180	7	6	1	3
30	1	3	3	1	6	3.875	3.12	3.12	32	371259	6	6	1	5
30	2	3	3.3	1.4	5.5	4.5	5	2.5	80	371259	6	3	1	4
31	2	1	2.1	1	4	3.375	4.55	0	22	65663	5	2	2	6
31	1	1	3	1.7	4.5	5.625	7.14	0	70	65663	7	7	2	2
32	2	2	2.3	1.1	5.6666667	4.625	5.17	0	58	328568	3	5	2	2
32	1	2	1.7	1.2	5.6666667	4.75	4.05	1.35	74	328568	5	5	2	4
33	2	3	3.6	1	6.5	6.25	8.89	0	45	218036	7	7	1	1
34	1	2	2	2	5.1666667	4.25	4.04	4.04	99	212424	6	3	2	1
34	2	2	3.7	1.6	5.3333333	4.25	3.77	0.94	106	212424	7	2	2	4
35	1	3	2.3	1.1	6.1666667	5.5	3.7	5.56	54	285868	7	NA	1	1
35	2	3	2.5	1	5	3.75	5.36	1.79	56	285868	6	NA	1	3
36	2	3	2.6	1.6	4.5	5	4.4	3.3	91	88868	6	5	1	6
36	1	3	3.3	1.3	5.1666667	4.75	8.33	0	108	88868	7	6	1	2
37	2	2	3.6	1.4	6.6666667	5.625	8.82	0.98	102	600000	6	5	2	4
37	1	2	3.3	1.1	5.6666667	3.875	3.21	1.28	156	600000	3	3	2	6
38	2	2	2.8	1.1	5.6666667	5.25	4.55	0	88	371259	6	3	2	3
39	1	1	1.6	1.2	5.1666667	4	2.27	6.82	44	110235	5	4	2	1
39	2	1	1.8	1	4.8333333	3.5	1.87	0.93	107	110235	2	2	2	1
40	1	1	3.6	1.2	5.6666667	4.75	3.7	1.23	81	600000	6	4	1	1
40	2	1	3.5	1.1	6	4.875	4.8	1.6	125	600000	5	5	1	1

41	1	1	1.8	1.3	5.6666667	3.875	2.2	0	91	199186	2		2	2	1
41	2	1	3.1	1.7	4.3333333	4.25	0	1.19	84	199186	6		5	2	4
42	1	1	2.5	1.6	4.6666667	4	5.63	2.82	71	120004	6		6	2	1
42	2	1	2.7	1.8	5.5	4.625	1.94	1.94	103	120004	6		3	2	3
43	2	1	3.1	1.1	5.6666667	4.875	6.25	0	64	342441	6		5	1	1
43	1	1	2.3	1.6	5.3333333	3.5	0	0	101	342441	6		3	1	1
44	2	1	2	1	5.8333333	5.5	1.65	0.83	121	164206	6		4	2	1
44	1	1	2.7	1.3	5.6666667	4.5	4.19	0.6	167	164206	6		4	2	1
45	2	2	1.7	1.1	6	4.875	0	0	88	336680	6	NA		2	2
45	1	2	2.4	1	5.6666667	4.75	2.86	0.95	105	336680	6	NA		2	1
46	2	2	3	1	5	5	2.47	1.23	81	201867	4		3	1	1
46	1	2	3.4	1.9	5.6666667	5.5	3.37	1.12	89	201867	7		3	1	4
47	1	2	3.8	1.1	5.8333333	5.375	4.17	0	96	503807	6		5	2	1
47	2	2	3.1	1.2	5.3333333	5.5	4.1	0.82	122	503807	5		2	2	4
48	1	2	2.9	1.2	6.8333333	3.875	3.3	0	91	134455	5		5	2	5
48	2	2	2.4	1.1	5.8333333	4.625	0.85	0	117	134455	6		5	2	6
49	2	2	2.8	1.4	5.1666667	4.75	4.88	1.63	123	21635	3		3	2	1
49	1	2	2.5	2.4	5.8333333	2.875	2.68	2.68	112	21635	5		2	2	1
50	2	2	2.2	1	5.6666667	5.25	6.06	0	33	407075	5		3	2	4
50	1	2	3.3	1.1	6.3333333	5.5	11.76	0	85	407075	4		2	2	6
51	1	3	3.4	1.1	7	5.5	7.02	0	57	220960	7		5	2	1
51	2	3	2.6	1.1	5.3333333	4.875	7.38	0.67	149	220960	7		5	2	2
52	2	3	2.9	1	5.8333333	3.25	5.26	1.75	57	183830	5		2	1	2
52	1	3	3.9	1.1	5.3333333	2.875	3.6	1.8	111	183830	7		3	1	1
53	2	3	2.9	1.2	5.3333333	4.625	1.37	1.37	73	547726	7		6	1	2
53	1	3	2.6	1	5.3333333	4	4	1.33	75	547726	7		2	1	4
54	2	3	3.2	1.1	5.8333333	5.125	3.16	1.05	95	108978	7		6	2	4
54	1	3	3.1	1.6	5.6666667	4.75	2.86	3.81	105	108978	7		6	2	1
55	1	3	3.2	1	6	5	5.17	1.72	58	88229	6		6	2	3
55	2	3	2.1	1.2	5.6666667	6.375	11.48	4.92	61	88229	7		6	2	1
56	2	3	1.9	1	5.6666667	5.125	3.03	1.52	132	600000	4		3	2	1
56	1	3	2.1	1.2	5.8333333	5.25	3.51	0.58	171	600000	6		5	2	1
57	2	1	2.6	1.8	6.5	3.375	4.17	2.78	72	311051	5		1	2	1
57	1	1	2.4	1.1	6	4.625	3.42	0	117	311051	4		2	2	1
58	1	2	2.4	1.2	4.8333333	4.25	3.33	0	90	42655	6		3	1	1
58	2	2	2	1.2	4	3.875	2.78	2.78	72	42655	4		3	1	2
59	2	1	2.2	1.6	3.5	4.25	2.8	2.8	107	149075	3	NA		2	1
59	1	1	1.2	1.5	4.1666667	3.5	3.85	1.28	78	149075	4	NA		2	1
60	2	3	3.5	1.1	6.3333333	5.5	4.23	0	71	319961	6		5	2	1
60	1	3	2.1	1.4	6.6666667	5.25	6.73	0.96	104	319961	6		6	2	1
61	1	2	3.4	1.8	6.6666667	4.75	1.67	1.67	60	194228	3	NA		2	1
61	2	2	1.4	1.3	4.8333333	3.75	3.9	2.6	77	194228	4	NA		2	1
62	1	1	1.3	1.2	4.8333333	5.25	2.6	0	77	600000	6		5	2	6
62	2	1	2	1.1	5.6666667	4.125	1.41	0	71	600000	5		4	2	1
63	2	3	4.2	1.1	6.6666667	6	2.2	1.1	91	230183	7		5	1	1
63	1	3	2.8	1.5	6.1666667	5.875	2.04	2.04	98	230183	7		3	1	1
64	1	3	3.1	1.3	5.8333333	5.375	2.42	0	124	123947	7		5	1	1
64	2	3	3.7	1	5.6666667	6	2.06	3.09	97	123947	6		7	1	1
65	1	1	4.2	1.2	6.3333333	5.5	5.11	1.46	137	52941	6		5	1	6
65	2	1	2.4	1.3	4.6666667	4.75	0	0	201	52941	6		6	1	4
66	2	3	3.8	1.6	6	6	2.48	0.83	121	244358	7		7	1	4
66	1	3	3.2	1.4	5.8333333	5.125	4.12	1.76	170	244358	6		6	1	5
67	1	1	4.7	1	7	6.375	4.23	0	71	600000	6		5	1	1
67	2	1	3.2	1.1	6.5	6	6.1	0	164	600000	6		5	1	1
68	2	2	3.3	1	5	4.875	2.5	0	40	99094	4		1	1	1
68	1	2	3.8	1	5.8333333	4.25	9.38	0	64	99094	6		3	1	1
69	1	2	2.1	1	4.8333333	3.75	1.32	0	76	157897	2		2	1	1
69	2	2	3.1	1.1	4.3333333	5.875	2.03	1.35	148	157897	6		5	1	1
70	2	1	2.7	1.4	5.5	4.125	3.3	1.1	91	600000	5		4	2	4
70	1	1	2.9	1.1	7	5.25	4.38	2.19	137	600000	3		1	2	1
71	1	1	3.6	1.6	6.5	4.875	3.57	0	56	166104	6		6	2	6
71	2	1	2.9	1.2	6.5	5	2.27	4.55	44	166104	5		5	2	1
72	1	3	4	1.5	6.1666667	4.875	6.82	0	44	445566	7		2	2	3
72	2	3	2.3	1.4	6	4.75	2.35	2.35	85	445566	7		3	2	4
73	1	2	2	1	5.1666667	4.5	4.82	3.61	83	292223	5		1	2	1
73	2	2	2.2	1.1	5.3333333	4.375	2.5	4.17	120	292223	6		3	2	4
74	1	3	2.6	1	6.5	5.125	8.82	0	68	224807	7	NA		2	1
74	2	3	3.8	1	5.8333333	5.5	6.52	1.09	92	224807	7	NA		2	6
